# Mechanisms Involved in Interspecific Communication between Wine Yeasts

**DOI:** 10.3390/foods10081734

**Published:** 2021-07-27

**Authors:** Ana Mencher, Pilar Morales, Jordi Tronchoni, Ramon Gonzalez

**Affiliations:** 1 Instituto de Ciencias de la Vid y del Vino (CSIC, Gobierno de la Rioja, Universidad de La Rioja), Finca La Grajera, Carretera LO-20, Salida 13, 26007 Logroño, Spain; ana.mencher@icvv.es (A.M.); pilar.morales@icvv.es (P.M.); 2 Faculty of Health Sciences, Valencian International University (VIU), C/Pintor Sorolla 21, 46002 Valencia, Spain; j.tronchoni@universidadviu.com

**Keywords:** wine yeast, interaction, communication, non-*Saccharomyces*

## Abstract

In parallel with the development of non-*Saccharomyces* starter cultures in oenology, a growing interest has developed around the interactions between the microorganisms involved in the transformation of grape must into wine. Nowadays, it is widely accepted that the outcome of a fermentation process involving two or more inoculated yeast species will be different from the weighted average of the corresponding individual cultures. Interspecific interactions between wine yeasts take place on several levels, including interference competition, exploitation competition, exchange of metabolic intermediates, and others. Some interactions could be a simple consequence of each yeast running its own metabolic programme in a context where metabolic intermediates and end products from other yeasts are present. However, there are clear indications, in some cases, of specific recognition between interacting yeasts. In this article we discuss the mechanisms that may be involved in the communication between wine yeasts during alcoholic fermentation.

## 1. Introduction

For several thousand years, the main step in the production of grape wine has been the spontaneous alcoholic fermentation of grape juice. This was driven by the natural epiphytic microbiota in grape berries, constituted by moulds, bacteria, and dozens of yeast strains, belonging to several non-*Saccharomyces* genera such as *Hanseniaspora*, *Candida*, *Debaryomyces*, *Starmerella*, *Dekkera*, *Kluyveromyces*, *Metschnikowia*, *Torulaspora*, *Pichia*, *Zygosaccharomyces*, *Cryptococcus* and *Rhodotorula* [1,2,3]. *Saccharomyces cerevisiae*, the species that would ultimately dominate the fermentation process, is usually present in much lower cell counts in healthy berries. Malolactic fermentation, a biotransformation process conducted by lactic acid bacteria, which takes place in most red and some white wines, usually after alcoholic fermentation has finished, is essential in many cases to achieve acceptable quality and stability [4]. It has also traditionally been a spontaneous process, and for that reason a source of uncertainty for winemakers. Historically, the main tools for the microbial management of alcoholic fermentation in cellars have been sulphiting agents. Wine isolates of *S. cerevisiae* tend to be particularly tolerant to sulphur dioxide compared to most other bacterial and yeast species in the same environment. This is due to specific genomic adaptations that seem to have been selected over centuries of domestication [5]. Combined with other traits, such as the Crabtree effect, low oxygen requirement, or tolerance to ethanol, heat, and osmotic stress, this contributes to making *S. cerevisiae* the dominant species in most non-inoculated fermentations. While these spontaneous processes can result in wines of excellent quality, with a great complexity of sensory nuances, they also lead to a high degree of uncertainty, given the relatively high incidence of microbial spoilage (driven by both yeast and bacteria), difficult starts, sluggish and even stuck fermentations.

The main qualitative improvement in the microbiological control of alcoholic fermentation took place during the last decades of the 20th century, with the stepwise popularisation of commercial *S. cerevisiae* starter cultures. Inoculation of wine strains of *S. cerevisiae* does not turn fermenting grape must into a bona fide microbial monoculture, but it brings it quite close to other biotechnological processes driven by a single species [6]. Under the perception of inoculated fermentations as virtually pure cultures, microbial interactions were of little interest to researchers and professionals. This lack of interest was not totally surprising, since most of our knowledge on the biology of microorganisms has been traditionally obtained through the study of pure cultures [7]. Nevertheless, there is a growing interest in the ecological context as a key to understanding yeast biology and evolution [8].

However, at the turn of the 21st century, our knowledge of the microbial ecology of alcoholic fermentation and the physiology of other wine yeast species was much better than a few decades earlier. There was also an increasing demand from marketing departments for product differentiation and diversification. Altogether, this led to alternative species (collectively known as non-*Saccharomyces*) entering the wine yeast starter market. They were primarily aimed at contributing to the aromatic complexity of wines while avoiding the risks of spoilage associated with spontaneous fermentation. In addition to this initial application, non-*Saccharomyces* yeasts are nowadays proposed to achieve many different technological objectives, including malic acid reduction, lactic acid production, volatile acidity control, alcohol level reduction, release of polysaccharides, or even as biocontrol agents against spoilage microorganisms [9,10,11,12,13,14].

Nevertheless, for most of these species, ethanol toxicity leads to metabolic arrest and cell death as fermentation progresses. Fermentation would eventually be taken over by *Saccharomyces* strains of the native microbiota, but very often this will happen after a period of sluggish fermentation. Therefore, to ensure complete fermentations, non-*Saccharomyces* starters are mostly used in combination with *S. cerevisiae*, either in co-inoculation or in sequential inoculation [12]. In either case, the coexistence of two yeast species, with relatively high cell densities, is a novelty compared to the use of pure cultures and has triggered interest in the study of starter culture interactions. Another situation in which microbial interactions between wine starters are becoming relevant is the growing practice of inoculating lactic acid bacteria from the early stages of fermentation, to carry out alcoholic and malolactic fermentation simultaneously [15]. However, in this article we will focus on the interactions between yeast species.

## 2. Microbial Interactions between Wine Yeasts

The study and classification of interactions between microbial species can be approached from different points of view. From an ecological perspective, different types of binary interactions can be distinguished, depending on their effect on the survival of each partner. The most widely accepted classification contemplates six options; mutualism (+/+), commensalism (+/0), neutralism (0/0), amensalism (−/0), antagonism based on parasitism (+/−) or predation (+/−), and competition (−/−). Where (+) indicates that a partner benefits from the presence of the other, (−) indicates that it is impaired, and (0) indicates that the interaction has no effect on it [16]. However, some authors consider that this classification fails to capture all the details that can shape interactions under real-life conditions [17]. During wine fermentation, competition seems to be the dominant mode of interaction between yeast species, although some lactic acid bacteria might show commensalism with yeasts [18]. In turn, competition between a pair of microorganisms can have exploitation or interference components [19]. Exploitation competition is clearly present in wine fermentation. Nitrogen sources, as well as some vitamins and trace elements, often become limiting during alcoholic fermentation [20,21]. Likewise, available oxygen is quickly consumed by yeasts, leading to anaerobic conditions, which are growth-limiting for many wine yeast species [22,23]. Even availability of carbon sources, that seem to be in far excess compared to other yeast nutrients, might contribute to the exploitation component of competition in some cases [24]. During wine fermentation, major fermentation metabolites such as ethanol, acetic acid, or carbon dioxide, reach concentrations that become disruptive or toxic to many yeasts. This contributes to interference competition [25,26,27].

**Figure 1:**
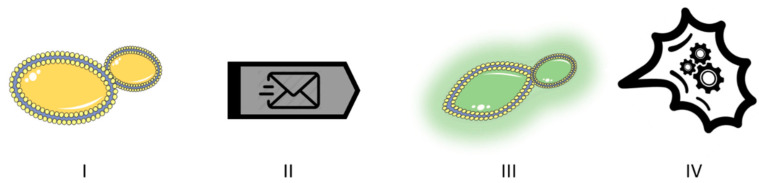
True yeast-to-yeast communication requires four elements. I. A transmitting yeast cell. II. The message (signal). III. A perceiving yeast cell. IV. A physiological response by the receiving cell.

In addition, we could distinguish interactions that involve a communication mechanism between the partners from those that do not. True yeast-to-yeast communication involves at least four elements (Figure 1): a transmitting yeast, a signal or message, a receiving yeast, and a physiological response or reaction from the receiving yeast. In a bidirectional communication, each of the species involved would act as both sender of a signal and receiver of the signal emitted by the other yeast. In any case, there is a wide grey area, considering that our knowledge of communication signals is still very limited. Therefore, we cannot establish watertight compartments between interactions that do or do not involve real communication. Table 1 shows examples of the different signal types described below.

## 3. Signal Types

### 3.1. Unspecific

Certainly, exploitation and interference competition must trigger response mechanisms linked, for example, to nutrient availability or abiotic stress [28]. Despite changes in the physico-chemical environment not being considered as part of a true communication process, they are nonetheless informative for the cell. As mentioned above, interference competition may rely on some major metabolic endpoints, but there are other broad-spectrum mechanisms for interference competition that are worth mentioning in this section (Table 1). The best known of these is the secretion of killer toxins [29,30]; even though, in nature, we can find both low and high-specificity killer toxins in different yeast species (see below). Yeast killer toxins were first discovered in *S. cerevisiae* but have now been described for over one hundred yeast species. They are very diverse in their interference mechanism, although many of them target the cell surface (plasma membrane or cell-wall). Known *S. cerevisiae* killer toxins are encoded by double-stranded cytoplasmic RNAs (packaged in virus-like particles). However, depending on the yeast species, killer toxins may also be encoded by other episomal elements or in nuclear chromosomes [31]. Killer positive *S. cerevisiae* wine yeast isolates are usually K2 or Klus [32].

Also, peptides derived from *S. cerevisiae* glyceraldehyde-3-phosphate dehydrogenase (GAPDH) show antimicrobial activity against different wine-related yeasts and bacteria [33,34,35]. These antimicrobial peptides (AMPs), also named as saccharomycin [36], seem to alter plasma membrane H^+^-ATPase or ATP permeability of target cells.

Finally, pulcherriminic acid is an iron chelating cyclopeptide secreted by several microorganisms (yeast and bacteria), giving rise to the red/brown pigment pulcherrimin when complexed with iron. It reduces iron bioavailability for competing microorganisms and is considered to play an important role in microbial ecology in different environments. Pulcherrimin was first described for *Metschnikowia pulcherrima* [37], a yeast species frequently found in winemaking environments, which is being developed as a non-*Saccharomyces* starter and as a biocontrol agent against wine spoilage yeasts [38].

### 3.2. Specific

#### 3.2.1. Contact-Dependent

A first approach to address whether two oenological yeasts establish specific interactions is to use culture devices that limit cell-to-cell contact, while sharing the same physico-chemical environment. Hollow fibre filters and semi-permeable membranes have been used for this purpose [39]. Population dynamics, fermentation kinetics or the profile of secondary metabolites are then compared with fully mixed cultures. To avoid drawing wrong conclusions, it is important in these cases to make sure that all soluble compounds are in equilibrium on both compartments [39]. Similar behaviour of the two yeast strains in both culture systems could rule out cell-to-cell contact to be involved in the observed interaction. Conversely, clearly different behaviour is indicative of a contact-dependent interaction. There are several recent publications on wine yeast interactions proposed to be contact-dependent [40,41,42], as well as contact-independent [43,44,45]. Although most examples of cell-to-cell interaction refer to interference competition, stimulatory effects have also been observed on occasion [41].

In *S. cerevisiae*, many of the cell surface properties and growth morphologies, including adhesion, depend on the expression and features of *FLO* genes, which encode or regulate the expression of flocculins [46,47]. Rossouw et al. [48] observed the formation of mixed flocs of *S. cerevisiae* strains, whether flocculating or not in pure culture, with yeasts of other species. These interactions depended differently on the expression of different *FLO* genes, which may favour or exclude co-flocculation with different species. These authors suggest that these properties have evolved as a way for yeasts to construct ecological niches. By manipulating the expression of *FLO* genes, they were able to demonstrate the importance of physical contact in ecosystems where several yeast species coexist [49].

An interesting case illustrates the ambiguity of interpretation of experiments aimed at identifying contact-dependent interactions. In joint cultures of *S. cerevisiae* with *T. delbrueckii*, *Lachancea thermotolerans*, or some other species there were both reports on interactions dependent on GAPDH-derived AMPs released into culture media by *S. cerevisiae* [33,34], and contact-based interactions [50,51]. Later, authors from the same research groups concluded that these were non-exclusive mechanisms [52]. Indeed, AMPs might be mediating both contact-independent and contact-dependent communication (Table 1), since they are accumulated and displayed on the surface of the producer cells [53].

Sometimes, cell-to-cell proximity can be complementary to interference mechanisms using soluble molecules. An example could be the one just mentioned, with AMPs presented on *S. cerevisiae* cell surfaces. However, also interesting is the case illustrated by Pérez-Torrado et al. [54] on the interaction between two strains of *S. cerevisiae*. These authors described a dominance phenomenon under alcoholic fermentation conditions, dependent on cell-to-cell contact, whereby the dominant strain generates interspecific aggregates along with an SO_2_-rich microenvironment, to which the other *S. cerevisiae* strain is more sensitive.

#### 3.2.2. Contact-Independent

In the absence of direct contact, communication between yeast cells can use signals of various types, including gases, other volatile molecules, low or high molecular weight soluble compounds, and probably more complex structures, such as extracellular vesicles. In this section we describe these signals in order of increasing molecular size.

During surface growth in some culture media, *S. cerevisiae* colonies produce ammonium pulses that serve to guide the growth of neighbouring colonies, resulting in minimisation of competition for available nutrients [55,56]. Since several yeast genera use similar signals, ammonium pulses might act on both intraspecific and interspecific communication. This phenomenon has been described for growth on surfaces, and may seem of little relevance during alcoholic fermentation, but it may be important in cellar surfaces or in the vineyard.

Acetaldehyde is a key intermediate of alcoholic fermentation; it influences the evolution of polyphenolic compounds during wine fermentation and aging [57] and combines reversibly with sulphur dioxide [58]. Acetaldehyde is released in significant amounts by *S. cerevisiae* and non-*Saccharomyces* wine yeast species during wine fermentation [59]. It has also been shown that extracellular acetaldehyde serves as a signal to synchronise glycolytic oscillations in *S. cerevisiae* cultures [60]. Glycolytic oscillations have only been observed under very particular culture conditions, but a role of acetaldehyde as a communication signal between yeast cells, under winemaking conditions, cannot be ruled out.

Other abundant, low molecular weight, organic molecules that have been involved in microbial communication mechanisms relevant for winemaking are acetic and lactic acid. Both have been claimed as inducers of the [*GAR^+^*] prion in *S. cerevisiae* and other fungi [61,62]. These metabolites can be produced in large quantities by lactic and acetic acid bacteria found in the winemaking ecological niche, and might then mediate inter-kingdom communication (see next section for details on the yeast response in terms of prion induction). Lactic acid is also produced in relevant amounts by some non-*Saccharomyces* wine yeasts, such as *L. thermotolerans*, during alcoholic fermentation [2].

Several aromatic alcohols, mainly those derived from tyrosine, tryptophan, and phenylalanine via the Ehrlich pathway, have been linked to quorum sensing mechanisms in yeasts [63,64]. Physiological responses related to quorum sensing depend on cell density, but also on the availability of nitrogen nutrients, which is a relevant constraint in winemaking. One of the most studied processes related to this are dimorphic transitions in *Candida albicans* and *S. cerevisiae* [65,66]. It has recently been shown that tryptophol, phenylethanol, and tyrosol, in addition to serotonin and tryptamine (derived from tryptophan), are able to induce changes in the growth and morphogenesis of various wine yeast species [67]. Interestingly, these effects are observed using concentrations of these compounds similar to those found during alcoholic fermentation. Therefore, it is conceivable that these molecules may play a signalling role under industrial production conditions, beyond the impact of some of them on sensory traits. Furthermore, it has recently been found that *S. cerevisiae* responds to typical bacterial quorum sensing molecules by modulating ethanol tolerance and cell morphology [68]. Conversely, several yeast species can inactivate bacterial quorum sensing signals [69]. This opens the possibility for not only quorum sensing, but also quorum quenching, playing a role in microbial interactions in winemaking.

Macromolecules can also constitute yeast-to-yeast communication signals. The paradigmatic case is the sex pheromones of *S. cerevisiae*. These are 12–13 residue peptides, with some post-translational modifications, which can induce drastic changes in cell physiology and gene expression patterns. Sensitive cells (i.e., haploid cells of the complementary mating type) stop the cell division cycle in the G1 phase and display oriented growth toward the mating partner [70]. Nonetheless, it seems that sporulation or sexual reproduction are not common in *S. cerevisiae* during alcoholic fermentation [71].

The largest structures potentially involved in signal transmission between yeasts are extracellular vesicles (EVs). EVs, ranging in size from 20 nm to 500 nm, appear to be produced by almost any type of living cell [72]. Their lumen is surrounded by a lipid bilayer, and they can carry proteins and various types of RNAs [73,74]. EVs are involved in various communication processes, both intra- and inter-specific [75]. In the case of fungi and yeasts, most of the available information regarding EVs refers to human, animal or plant pathogenic species [76]. EVs have also been investigated in *S. cerevisiae*, providing further insight into the mechanisms of biogenesis and possible functions [77,78]. Regarding the oenological environment, the production of extracellular vesicles in six different wine yeast species has recently been described, and the protein composition of EVs from *S. cerevisiae* and *Torulaspora delbrueckii* has been characterised [79]. Both were shown to be enriched in glycolytic enzymes and cell-wall related proteins. Indeed, the most abundant protein in the EV-rich fraction of these two species, as well as *L. thermotolerans*, is exo-1,3-β-glucanase. Demonstration of EV production under alcoholic fermentation conditions, and its relationship with communication processes in other contexts, has led to the proposal that EVs could be involved in signalling processes also during winemaking [80]. Related to this, studies focusing on contact-based interactions between cells have found interactions both dependent and independent of physical separation (see above). There is a variety of devices and cut-off sizes used that would differently restrict EVs transfer between compartments. However, none of these studies addressed the question of the involvement of EVs in the interaction (or their passage through the separation system). It would be interesting to design similar devices focusing on the transfer of EVs, and to further investigate the composition of wine yeast EVs (e.g., nucleic acids or lipids) to find out more about the role of EVs in the communication processes in this context.

Finally, we should bear in mind that our current view on some of the low-specificity signals described above may be challenged by new discoveries and hypotheses. For instance, an increasing number of killer toxins are being discovered in many different yeast species, including both broad-spectrum and highly specific toxins [29,30]. Some authors have suggested that killer toxins at sub-inhibitory concentrations may act as communication signals rather than inhibitory molecules [30]. A similar possibility had been previously proposed for the ecological and evolutionary role for antibiotics in natural environments [81,82]. Similarly, we can imagine a signalling function for *S. cerevisiae* antimicrobial peptides derived from GAPDH. On the other hand, pulcherrimin has been involved in the coordination of biofilm growth arrest in *Bacillus subtilis* [83]. Again, an intriguing possibility is pulcherrimin behaving as a signalling molecule in wine ecosystems involving *M. pulcherrima* (an important pulcherrimin producer). The role of some of these yeast toxins in intercellular communication warrants further investigation.

## 4. Cell Responses to Communication Signals

Yeast physiological responses can develop through a variety of interrelated mechanisms, so that most studies can provide only a partial picture of the biological processes involved. Moreover, as pointed out by Shekhawat et al. [84], the use of long co-culture times, in many of the works addressing microbial interactions in winemaking, makes it impossible to distinguish specific responses from the indirect ones. This is generally the case for studies assessing population dynamics or fermentation kinetics. Sequential inoculations are a clear example of the difficulty of attributing changes in gene expression, after hours or days of growth of the first yeast species, to a specific response rather than to changes in the environment [85,86].

In some cases where physical interaction is involved, the resulting structure (floc, biofilm, flor) is the most obvious result of the interaction [47,87]. On other occasions, the interaction results in changes in cell morphology, such as the polarised growth induced by *S. cerevisiae* pheromones on recipient cells, or the hyphal or pseudohyphal growth of several wine yeast species induced under certain circumstances by aromatic amino acid derivatives [67]. However, not every time physical contact is involved in yeast interactions does it lead to the formation of a particular physical structure or cell morphology.

The most specific interactions, among those presented so far, have been often revealed under experimental conditions far removed from oenological practice. Examples are growth in solid media, or in media with a composition quite divergent from that of grape must. For this reason, their relevance in “real life” requires further research. However, we should not lose sight of the fact that, even in conditions closer to natural environments, the physico-chemical context can have an important impact on yeast-to-yeast interactions. For instance, under low oxygen conditions, *L. thermotolerans* and *T. delbrueckii* decrease their coexistence times in mixed cultures with *S. cerevisiae* [24,88]. The aeration regime also conditions the output of fermentation experiments co-inoculated with *M. pulcherrima* and *S. cerevisiae* [22]. Finally, the abundance of anaerobic growth factors seems to condition the physical interaction between *T. delbrueckii* and *S. cerevisiae* cells, while growth temperature influences the interaction between *L. thermotolerans* and *S. cerevisiae* [41,89].

Intracellular events in response to different external stimuli often involve mitogen-activated protein kinase (MAP kinase)-mediated signal transduction cascades, which ultimately lead to transcriptional reprogramming [90]. Indeed, one of the best-known model systems of such signalling pathways is the response of haploid *S. cerevisiae* cells to mating pheromones [91,92]. However, regarding the response to specific interactions between oenological yeasts, our current knowledge is far from that level of detail. Milanovic et al. [93] observed changes in *ADH1* and *PDC1* expression in *S. cerevisiae* in the presence of immobilised *Starmerella bombicola* cells. These changes were related to those in wine composition. Subsequently, research from our group described global transcriptional responses of *S. cerevisiae* to the presence of up to four non-*Saccharomyces* yeast species after two or three hours of co-culture in synthetic must [94,95,96]. This early response suggests a specific reaction to co-cultivation, not simply caused by changes in nutrient availability, for example. The responses of *S. cerevisiae* are differentiated depending on the other oenological yeast species in co-culture, although there are some common features, suggesting a stimulation of metabolic activity [95,96] which could be evidenced experimentally [94]. In the case of *T. delbrueckii*, a specific response to co-culture was also observed, although it was delayed relative to *S. cerevisiae*. Alonso del Real et al. [42] also found a faster transcriptional response of *S. cerevisiae* to co-cultures in mixed cultures with *S. kudriavzevii*. It seems that an early transcriptional response to co-culture is a distinctive feature of oenological strains of *S. cerevisiae*. Pérez-Torrado et al. [54] found changes in the transcriptome due to intraspecific competition between two *S. cerevisiae* strains. In these cultures, one of the strains was clearly dominant over the other. Transcriptional changes were much more marked in the non-dominant strain, but the dominant strain showed consistently higher activation of *SSU1* and genes coding for some surface proteins, which the authors relate to the dominance mechanism (see above). Shekhawat et al. [84] used continuous cultures to avoid the masking effect of general changes in medium composition in the study of specific interactions between *S. cerevisiae* and *L. thermotolerans*. They observed an interaction between environmental conditions (aerobic or anaerobic) and co-culture on the transcriptomic response of both species. Some of the differentially expressed genes suggest that cell-to-cell contact might play a relevant role in the interaction.

In addition to morphological, transcriptional, and metabolic reprograming, some microbial interactions in wine might involve long term epigenetic changes, as illustrated by the induction of a [*GAR*^+^] prion in *S. cerevisiae* by lactic or acetic acid bacteria. [*GAR*^+^] is a protein-based heritable element that partially relieves carbon catabolite repression [97]. Induction of [*GAR*^+^] is proposed to be advantageous for both bacteria and yeasts sharing the same environment. Bacteria would benefit from a lower ethanol production by *S. cerevisiae* [98], while *S. cerevisiae* would receive easier access to alternative carbon sources [97]. Similar elements have been described in a range of yeast species [99]. However, although prion states tend to be relatively stable, [*GAR*^+^] shows low phenotypic penetrance [100], so that only a handful of the prion-positive cells would benefit of any hypothetical advantage.

## 5. Conclusions

Wine is the result of the activity of many different microorganisms, and this activity is probably modulated by the interactions established between them. Interest in these interactions, as a research topic, has been boosted by the entry into the market of non-*Saccharomyces* wine starter cultures. Many different works have shown that yeast-to-yeast interactions during wine fermentation do exist, and they can become quite complex when considering an increasing number of initial yeast species [101,102,103]. In many instances, it is possible to establish correlations between the physiological properties of the strains and the population dynamics or metabolite profiles in a mixed fermentation. However, there are levels of interaction for which our knowledge is still very scarce. In this article we have focused on the interaction mechanisms that involve the exchange of specific signals among the yeast partners. We have tried to describe current knowledge on the nature of such communication signals and the biological responses of yeast cells to them. There is a wide variety of molecules than can become communication signals between yeast species. It is also worth noting that responses to these signals can be dose-dependent, so that molecules considered as toxic or growth-limiting at a given concentration might induce a different biological response at lower concentrations. The field of biological interactions between wine starter cultures is just starting and will probably still produce many interesting surprises. Some of them would certainly bring about practical implications for the design of mixed wine starter cultures and the control of industrial wine fermentation.

## Figures and Tables

**Table 1:** Examples of communication mediators grouped by signal type.

Signal Type	Interaction Mechanism	Mediator Examples
Unspecific		Nutrient availability Major metabolic endpoints (e.g., ethanol) Broad specificity killer toxins Antimicrobial peptides (GAPDH derived) Pulcherriminic acid
Specific	*Contact-dependent*	Flocculins Cell-wall associated antimicrobial peptides
	*Contact-independent*	Ammonium Acetaldehyde Quorum sensing molecules Sexual pheromones Extracellular vesicles
